# Practice Tools for Screening and Monitoring Insomnia in Children and Adolescents with Autism Spectrum Disorder

**DOI:** 10.1007/s10803-021-05236-w

**Published:** 2021-08-31

**Authors:** Tobias Banaschewski, Oliviero Bruni, Joaquin Fuentes, Catherine Mary Hill, Allan Hvolby, Maj-Britt Posserud, Carmen Schroder

**Affiliations:** 1 Department of Child and Adolescent Psychiatry and Psychotherapy, Medical Faculty Mannheim, Central Institute of Mental Health, University of Heidelberg, Mannheim, Germany; 2 Department of Developmental and Social Psychology, Sapienza University, Rome, Italy; 3 Service of Child and Adolescent Psychiatry, Policlínica Gipuzkoa and GAUTENA Autism Society, San Sebastián, Spain; 4 School of Clinical Experimental Sciences, Faculty of Medicine, University of Southampton, Southampton, UK; 5 Department of Sleep Medicine, Southampton Children’s Hospital, Southampton, UK; 6 Department of Child and Adolescent Psychiatry, Psychiatry in Region of South Denmark, Esbjerg, Denmark; 7 Department of Regional Health Research, University of Southern Denmark, Odense, Denmark; 8 Department of Clinical Medicine, Faculty of Medicine, University of Bergen, Bergen, Norway; 9 Department of Child and Adolescent Psychiatry & Excellence Centre for Autism and Neurodevelopmental Disorders STRAS&ND, Strasbourg University Hospitals & University of Strasbourg Medical School, 67000 Strasbourg, France; 10 CNRS UPR 3212, Institute for Cellular and Integrative Neurosciences, Strasbourg, France; 11 Sleep Disorders Center& International Research Center for ChronoSomnology, Strasbourg, France

**Keywords:** Child Adolescent Sleep Insomnia Autism spectrum disorder ASD

## Abstract

Between 50–80% of children with autism spectrum disorder (ASD) have insomnia, which adversely affects their mental and physical health. However, there is no consensus to-date on suitable tools for insomnia screening and monitoring in daily clinical practice. An expert panel of child neuropsychiatry and sleep specialists, with expertise in children with neurodevelopmental disabilities, recommends: (1) performing insomnia screening of all children with ASD; (2) considering discussion or referral to a sleep specialist when comorbid sleep disorders are suspected. The panel further developed structured, brief screening and monitoring tools to facilitate insomnia screening and management in daily practice, monitor treatment effectiveness and standardize and compare outcomes across clinical settings to improve care and well-being of children with ASD and their families.

Sleep is essential for optimal child development and health. According to consensus statements on the recommended sleep duration by age, children and adolescents need more sleep than adults (Hirshkowitz et al., 2015; Morgenthaler et al., 2006; Paruthi et al., 2016). Inadequate and disrupted sleep can have detrimental effects on cognition (memory, attention, overall IQ), daytime behavior, and even language acquisition in the developing child (Dionne et al., 2011; Gruber et al., 2011; Kocevska et al., 2017; Sadeh, 2007; Vriend et al., 2012). Uninterrupted night sleep is optimal for the plasticity changes needed for learning and memory consolidation (Maski et al., 2015). The duration of uninterrupted sleep (longest sleep episode; LSE) evolves rapidly in early childhood, to reach a minimum of 6 consecutive hours of uninterrupted sleep at night by 17 months of age (Touchette et al., 2005). Healthy sleep has daytime benefits in typically developing (TD) children (Gordon, 2000), in children with developmental disorders (Wasdell et al., 2008) and in children with autism spectrum disorder (ASD) (Minde et al., 1994).

ASD is characterized by impairment in social interaction/communication and repetitive, restrictive, or unusual sensorimotor behaviors and interests (DSM-5, 2013a, 2013b). It affects 1/54 live births according to the Centers for Disease Control and Prevention (CDC) (https://www.cdc.gov/ncbddd/autism/data.html), and around 1/160 of all children worldwide, according to the World Health Organization (WHO) (https://www.who.int/news-room/fact-sheets/detail/autism-spectrum-disorders) and numbers are growing. ASD can be diagnosed as young as 18 months of age, and effective interventions may improve developmental trajectories (Hyman et al., 2020). Therefore, many agencies recommend standardized screening for ASD at 18 and 24 months of age with ongoing developmental surveillance in pediatric primary care (although it may be performed in other settings). Physicians should be familiar with the diagnostic criteria for ASD, appropriate functional and etiologic evaluation, and co-occurring medical and behavioral conditions (such as disorders of sleep and feeding, gastrointestinal tract symptoms, obesity, seizures, attention-deficit/hyperactivity disorder, anxiety) that affect the child’s function and quality of life (Hyman et al., 2020). Insomnia is a major co-occurring condition in children with ASD (Goodlin-Jones et al., 2008; Miano et al., 2007; Richdale, 1999) and may precede ASD diagnosis (Humphreys et al., 2014; MacDuffie et al., 2020).

The Diagnostic and Statistical Manual of Mental Disorders-5th edition (DSM-5) (DSM-5, 2013a, 2013b) defines insomnia disorder as a predominant complaint of dissatisfaction with sleep quantity or quality (Table 1). DSM-5 does not distinguish specific ages or age ranges when defining the diagnostic criteria for insomnia disorder. In children with ASD the prevalence of insomnia is within the range of 41–86% (Goodlin-Jones et al., 2008; Miano et al., 2007; Richdale, 1999) (up to 10 times more frequent as compared to their TD peers) and tends to persist (Lai et al., 2019; Richdale, 1999). According to the literature, the insomnia in ASD is characterized mainly by difficulty maintaining sleep (34% mid-sleep awakenings and/or early awakenings) and difficulty initiating sleep (26%) (Krakowiak et al., 2008; Taira et al., 1998) which result in shorter sleep duration, with no evidence of an association between age and sleep disturbance (Katz et al., 2018).

**Table 1:** DSM-5 diagnostic criteria of insomnia disorder 307.42 (F51.01)

Criterion	Description
A	A predominant complaint of dissatisfaction with sleep quantity or quality associated with one (or more) of the following symptoms: 1) Difficulty initiating sleep (in children, this may manifest as difficulty initiating sleep without caregiver intervention), 2) Difficulty maintaining sleep, characterized by frequent awakenings or problems returning to sleep after awakenings (in children, this may manifest as difficulty returning to sleep without caregiver intervention or 3) Early-morning awakening with inability to return to sleep
B	The sleep disorder causes clinically significant distress or impairment in social, occupational, educational, academic, behavioral, or other important areas of functioning
C	The sleep difficulty occurs at least 3 nights per week
D	The sleep difficulty is present for at least 3 months
E	The sleep difficulty occurs despite adequate opportunity for sleep
F	The insomnia is not better explained by and does not occur exclusively during the course of another sleep–wake disorder (e.g., narcolepsy, a breathing-related sleep disorder, a circadian rhythm sleep–wake disorder, a parasomnia)
G	The insomnia is not attributable to the physiological effects of a substance (e.g., a drug of abuse, a medication)
H	Coexisting mental disorders and medical conditions do not adequately explain the predominant complaint of insomnia

Chronic sleep disturbances experienced by children with ASD are less likely to remit with age, and continue even into adulthood (Humphreys et al., 2014; Jones et al., 2016). Co-occurring sleep problems in children with ASD predicted more severe autistic core symptomatology (Schreck & Richdale, 2011), later development of attention-deficit/hyperactivity disorder (ADHD) symptoms in younger children and somatic complaints in older children (Hvolby et al., 2020; Mazurek et al., 2019), irritability, aggressive behavior, regression, stereotypies and anxiety (Uren et al., 2019; Yavuz-Kodat et al., 2020) poor cognitive (memory, attention, overall IQ) and performance and delayed language acquisition (Goldman et al., 2011; Katz et al., 2018; Veatch et al., 2017). Children with ASD who have longer sleep onset latencies (SOL) were more likely to have behavioral (e.g. bedtime resistance), psychological disturbances (sleep anxiety) or circadian misalignment (Inthikoot & Chonchaiya, 2020; Kang et al., 2020). Shorter bouts of continuous sleep (LSE < 6 h) were associated with more irritability and stereotypic behavior in children with ASD (Yavuz-Kodat et al., 2020). Additional reports link chronic sleep disturbance with increased risk of physical health problems, including changes in cardiovascular, immune, endocrine, nervous system function, and poorer overall health-related quality of life in children with ASD (Delahaye et al., 2014; Haney & Kott, 2014).

Disturbed sleep in a child has a negative impact on the whole family’s health and well-being; inter alia it is associated with high levels of caregiver stress, maternal depression and family disorganization and impairs their proper employment or further education (Devnani & Hegde, 2015; May et al., 2015). Therefore, early identification and intervention may help to offset some of the potentially detrimental effects subsequent to prolonged sleep inadequacy in the developing child with ASD and their parents (Moore et al., 2017).

Despite the high prevalence of and morbidity associated with pediatric insomnia, sleep disorders in children often go undetected and untreated (Meltzer et al., 2010). Medical practitioners do not ask systematically about sleep concerns or parents do not seek assistance (Blunden et al., 2004) even though patients perceive that sleep issues per se are among the top health challenges of autism (McConachie et al., 2018; Morris et al., 2015). Additionally, parents and medical practitioners often have insufficient knowledge on sleep development and sleep problems (Schreck & Richdale, 2011). This is particularly problematic for children with ASD, as parents may present to the pediatrician with concerns regarding aggression, impulsivity, inattention/hyperactivity, or other behavioral disturbances that may be secondary to, or exacerbated by, a comorbid sleep disorder.

A clinical practice pathway, developed by the Autism Treatment Network (ATN) in association with the National Initiative for Children’s Healthcare Quality (NICHQ), emphasized the need for screening of sleep problems in ASD when seen by a non-sleep specialist (e.g. general pediatrician, primary care provider, child and adolescent neuro-psychiatrist, or autism medical specialist) (Malow et al., 2012). However, implementation of this pathway is challenging; only few physicians screen and/or document sleep problems during well-child visits (Mindell & Owens, 2003; Owens et al., 2003) and parents do not always accurately identify the properties/nature of sleep problems in their children with ASD (Honomichl et al., 2002). Physicians will generally ask the parent only a single question about their child’s sleep (Honaker & Meltzer, 2016) but caregiver response to a single question may miss the child’s sleep problem (Marvin et al., 2021).

Full insomnia diagnosis and evaluation requires expertise, time and resources while physicians have limited time per patient in which evaluation of various co-existing conditions related to ASD is required. More structured assessments, such as use of standardized questionnaires, can provide better documentation of the presence, severity and nature of sleep problems. However, many of the current tools are complex and the logistics of having to complete a questionnaire, have it appropriately scored, and then interpreting the results, tend to deter many physicians from this activity. To facilitate insomnia screening and treatment in this population, a panel of European sleep and neurodevelopmental experts has established a consensus on brief and efficient screener for sleep problems, and a structured tool to monitor insomnia treatment success that is short and easily implementable in daily practice in children and adolescents with ASD when seen by a non-sleep specialist.

## Insomnia Screening Tools

Because of social communication deficits and frequent developmental limitations most of the information about sleep in children with ASD is gained from parental report. The most frequent sleep problems in ASD, derived from parent completed questionnaires and sleep diaries, include sleep-onset insomnia, sleep-maintenance insomnia with long duration of nocturnal awakenings, and irregularities of the sleep–wake cycle, including early morning awakenings (Richdale, 1999; Williams et al., 2004). However, while understudied, medically based sleep disturbances beside insomnia, such as sleep disordered breathing, parasomnias, circadian rhythm disorders and movement disorders, most likely occur at a frequency similar to or potentially higher rate in children with ASD compared to the TD pediatric population (Malow et al., 2006). Identification of these problems is important, given the secondary consequences; for example, sleep disordered breathing (e.g., obstructive sleep apnea), can mimic symptoms of ADHD or other disruptive behavior disorders in TD children. If the presence of another or additional sleep disorder is suspected, a discussion or referral to a sleep expert is to be considered.

In routine medical care screening is ideally achieved using a validated tool for insomnia and guidance for discussion and/or referral to sleep specialists in case of suspected other comorbid sleep problems (e.g. sleep disordered breathing, sleep related seizures, parasomnias and movement disorders). There is a place for the use of objective measurements in the assessment of sleep disorders in ASD but all have some limitations in insomnia and in particular in this population. Actigraphy, has recently been validated in children with ASD (Yavuz-Kodat et al., 2019) but may be poorly tolerated in this population because of sensory challenges, including tactile defensiveness (Fawkes et al., 2015; Gringras et al., 2017; Souders et al., 2009). Polysomnography (PSG) which involves the recording of multiple physiological through sensors attached to the scalp and face (Miano et al., 2007) at a sleep specialist clinic, is considered the “gold standard” in the assessment of certain sleep problems. PSG is not recommended for insomnia diagnosis and presents even more challenges for children with ASD, who are likely to present with sensory hypersensitivity that make tolerating the sensors difficult (Malow et al., 2006). Nevertheless, PSG can identify problems that are less reliably detectable by other measures, including problems specific to stages of sleep, seizures, parasomnias, movement disorders or sleep disordered breathing (Hodge et al, 2012; Leu et al., 2011; Miano et al., 2007).

The most practical tools in the study of insomnia in children with ASD generally include parent-report questionnaires or rating scales, or completion of a sleep diary. The consensus panel advocated the following main properties of an optimal insomnia screening tool for daily practice:

- Valid for the screening of insomnia in TD children and applicable to children with ASD.
- Quickly implemented and not too extensive
- Having clear cutoffs discriminating insomnia/absence of insomnia for easy decision making in the clinic.
- Suitable for parents/caregivers or patients to complete before the visit, potentially through online platforms (optional)
- Should include a brief screening of other sleep problems and provide recommendations for referrals to sleep specialists when needed.

Although insomnia diagnostic criteria specify that there should be a behavioral consequence of the lack of sleep (Criterion B, Table 1) (DSM-5, 2013a, 2013b), behavioral challenges associated with sleep problems in TD children may actually be part of the basic pathology in ASD. It is therefore neither necessary nor recommended to identify at the screening stage whether the behavior is a cause or effect of the sleep problem in a child with ASD.

None of the existing questionnaires that have been used to assess sleep in pediatric populations matched the criteria for an optimal screening tool for daily use in the clinic so that all children, very early in their ASD diagnostic pathway, could be screened for sleep problems as an important comorbidity to ASD (Humphreys et al., 2014). The following three tools -CSHQ -Children's Sleep Habits Questionnaire; CSDI-Composite sleep disturbance index; and SDSC -Sleep Disturbance Scale for Children, were evaluated for insomnia screening because they were the most widely used questionnaires adopted for children, had some psychometric validation in children with ASD, had clear cutoffs to distinguish insomnia and found appropriate for use by caregivers/parents or patients (where applicable).

### The Children’s Sleep Habit Questionnaire [CSHQ; (Owens et al., 2000)]

The Children’s Sleep Habit Questionnaire (CSHQ) is a comprehensive screener of sleep disorders in children. The CSHQ was originally developed to assess sleep problems in TD children aged 4 to 10 years old, based on the pediatric International Classification of Sleep Disorders. However, it has been used to assess sleep disturbances in children of other ages and diagnostic populations, including children with ASD (AlBacker & Bashir, 2017; Goldman et al., 2011; Hodge et al., 2012; Malow et al., 2006; Souders et al., 2009; Yavuz-Kodat et al., 2020). The CSHQ produces a total score based on 45 items, and individual subscale scores based on 33 items, with higher scores indicating more severe sleep disturbance. The 33-item scale consists of eight subscales assessing bedtime resistance, sleep onset delay, sleep duration, sleep anxiety, night wakings, parasomnias, sleep disordered breathing and daytime sleepiness. Johnson and colleagues (Johnson et al., 2016) found in a sample of children with ASD (age 2–10 years) adequate psychometric properties of a modified version with five subscales assessing sleep routine problems, insufficient sleep, sleep-onset association problems, parasomnias/sleep-disordered breathing, and sleep anxiety. The authors indicated the relevance of the first three subscales for children with ASD, and also suggested the inclusion of additional items that would be particularly relevant to children with ASD such as disruptive behavior, rigidity, ritualistic behaviors and sensory arousal at bedtime, specific sleep-onset association problems, delayed sleep onset, and sleep anxiety (Johnson et al., 2016). A recent abbreviation of the CSHQ for use in the ASD population (Katz et al., 2018) reduced it to 23 items. However, the CSHQ is a rather extensive questionnaire even in its shortest version and the need to score multiple sub-scales and a complex scoring algorithm make it less practical in the clinic.

### The Composite Sleep Disturbance Index (CSDI)

The CSDI is a validated tool scoring the frequency and duration of sleep problems reported by parents (Appleton et al., 2012) and takes approximately five minutes to complete. The CSDI scores the frequency and duration of six sleep habits over the previous month (settling at bedtime, sleep induction, waking up during the night, resettling, early wake time and co-sleeping with caregivers). Each of the six sleep habits are scored from 0 to 2 and the total CSDI score range is 0 to 12. A score ≥ 4 indicates a severe sleep problem (Marvin et al., 2021). A seventh, non-scored, question on the CSDI asks the parent “How satisfied are you with your child's current sleep pattern?” with responses scored on a 5-category Likert scale. CSDI is normed down to 3 years of age. A significant correlation was found between CSDI score and sleep parameters assessed through parent reported diaries in subjects aged 2–18 with ASD (Gringras et al., 2017; Malow et al., 2021; Maras et al., 2018).

A recent U.S.-based study with 610 dyads of parents and children with ASD (mean (SD) age of 12.1 (3.6) range 3–17 years) showed a positive correlation between parent dissatisfaction from child sleep and CSDI score (Marvin et al., 2021). Severe sleep problems (CSDI ≥ 4) had serious implications for child medical support and parent health, with subsequent significant economic burden on the family, (Coury et al., 2019) and more severe behavioral problems (Bennett et al., 2019). Drawbacks of the CSDI include a lack of defined criteria for evaluating sleep length and it does not include any items on other sleep disorders that should call for discussion or referral to a sleep expert clinic.

### The Sleep Disturbance Scale for Children (SDSC) (Bruni et al., 1996)

The SDSC is a 26-item scale developed to assess the presence of sleep difficulties in children within the previous six months and found to discriminate between controls and four clinical groups (insomnia, hypersomnia, respiratory disturbances during sleep and parasomnias). It is completed by the parent in approximately 5–10 min and useful in screening for sleep disturbances of school-age children (aged 6.5–15.3 years) in clinical and nonclinical populations. Notably, the SDSC has been used to measure sleep difficulties in children with disorders including ASD, ADHD, epilepsy and obsessive–compulsive disorder (OCD) (Jaspers-Fayer et al., 2018; Mancini et al., 2020; Marriner et al., 2017; Neto & Nunes, 2017; Precenzano et al., 2017) and was the only questionnaire to be ranked in the highest category for well-established evidence-based quality criteria by two reviews of pediatric sleep questionnaires (Lewandowski et al., 2011; Spruyt & Gozal, 2011). The SDSC section on ‘disorders of initiating and maintaining sleep (DIMS)’ includes seven questions rated on a 5-point Likert scales, from 1 (‘never’) to 5 (‘always [daily]’) (Table 2 part A). The DIMS section is short, intuitive and easily filled in by parents and can thus serve as a practical tool for daily screening of insomnia in the clinical setting. A DIMS score of 17 and higher is indicative of insomnia. However, the DIMS scale does not include any items on other sleep disorders that should call for discussion or referral to a sleep expert clinic.

**Table 2:** A novel screening tool for insomnia in children and adolescents (modified DIMS) (Bruni et al., 1996)

Part A: Insomnia Diagnosis: Child’s sleep habits in the last 3 months		Score
Date	• Child’s name	Age
The Child goes to bed reluctantly	• Never (1) • Occasionally (1–2 per month or less (2) • Sometimes (once or twice per week) (3) • Often (3–5 times per week (4) • Always (daily) (5)	
The child has difficulty getting to sleep at night	• Never (1) • Occasionally (1–2 per month or less (2) • Sometimes (once or twice per week) (3) • Often (3–5 times per week (4) • Always (daily) (5)	
The child feels anxious or afraid when falling asleep	• Never (1) • Occasionally (1–2 per month or less (2) • Sometimes (once or twice per week) (3) • Often (3–5 times per week (4) • Always (daily) (5)	
The child wakes up more than twice per night	• Never (1) • Occasionally (1–2 per month or less (2) • Sometimes (once or twice per week) (3) • Often (3–5 times per week (4) • Always (daily) (5)	
After waking up in the night, the child has difficulty to fall asleep again	• Never (1) • Occasionally (1–2 per month or less (2) • Sometimes (once or twice per week) (3) • Often (3–5 times per week (4) • Always (daily) (5)	
How many hours of sleep does your child get on most nights?	• 9–11 h (1) • 8–9 h (2) • 7–8 h (3) • 5–7 h (4) • Less than 5 h (5)	
How long, after going to bed, does your child usually fall asleep?	• Less than 15 min (1) • 15–30 min (2) • 30–45 min (3) • 45–60 min (4) • More than 60 min (5)	
Total Score (sum of subscale scores) Score 10 or lower: unlikely to have insomnia Score 11–16: at risk of having insomnia Score 17 or higher: insomnia		

Part B: Discussion/Referral to sleep specialist:		Score
The Child shows one or more of the following: • startles or jerks parts of the body while falling asleep and/or while asleep • often changes position • kicks the covers off the bed • shows repetitive actions such as rocking or head banging while falling asleep	• Never (1) • Occasionally (1–2 per month or less (2) • Sometimes (once or twice per week) (3) • Often (3–5 times per week) (4) • Always (daily) (5)	
The child snores loudly and/or has difficulty breathing during the night	• Never (1) • Occasionally (1–2 per month or less (2) • Sometimes (once or twice per week) (3) • Often (3–5 times per week (4) • Always (daily) (5)	
The Child shows one or more of the following: wakes up from sleep screaming or confused sleep walks has recurrent nightmares	• Never (1) • Occasionally (1–2 per month or less (2) • Sometimes (once or twice per week) (3) • Often (3–5 times per week (4) • Always (daily) (5)	
Score 3 or higher on any item: consider discussion and or referral to sleep specialist	Number of scores ≥ 3	

### A Novel Composite Screening Tool for Insomnia Based on Expert Panel Consensus (Modified DIMS) (Bruni et al., 1996)

The panel considered the DIMS section in the SDSC as a potentially useful screening tool for insomnia, but lacking a brief screening of other sleep problems to provide recommendations for referrals to sleep specialists when needed. Therefore, in the Modified DIMS proposed here some items of the SDSC related to the existence of other sleep disorders were condensed and included to signpost optional referral to a sleep expert clinic. The diagnosis of comorbid sleep problems does not preclude the presence of insomnia. The Modified DIMS tool (Table 2) proposed by the expert panel based on the SDSC (Bruni et al., 1996) is thus a simple screening tool that can be widely applied, early in ASD diagnosis. It is not intended to serve as the sole source of guidance in the evaluation of insomnia in children who have ASD or to replace clinical judgment and may not provide the only appropriate approach to this challenge. The intention of this clinical practice tool is to facilitate rapid screening of insomnia in ASD. This process should provide a framework for decision making related to best practices in the care of children and adolescents with ASD when seen by a variety of medical specialties in the non-sleep expert setting.

Part B of this tool can also be used in conjunction with the CSDI screener instead of the modified DIMS part A (insomnia screener).

## Insomnia Monitoring Tools

Following a positive screen for insomnia, the physician will consider therapeutic strategies. Therapeutic interventions should begin with parent education on good sleep–wake habits and the use of behavioral therapy as a first-line approach; if failed, pharmacological treatment may be indicated (Buckley et al., 2020). Clinicians should assure timely follow-up to monitor progress and resolution of insomnia to evaluate effectiveness of the therapy and its maintenance. Therefore, a structured evaluation tool for monitoring treatment status, treatment goals or success, which is also applicable for daily practice, is required. As children with ASD are referred to and treated by various physicians and specialties the alignment between practices is important for their optimal quality care. Yet another important reason for including standardized monitoring tools is that relying merely on parental perception of child’s sleep improvement (*has your child*’*s sleep improved*?) is insufficient because parents are exhausted, might have low expectations and be satisfied after minimal improvement. As a result, the clinician may miss further adjustments to optimize treatment.

Available monitoring tools are not intuitive, and require time-consuming guidance for parents (e.g. sleep diaries) or scoring of multiple sub-scales. There is no concise structured treatment evaluation tool, no clear evaluation criteria are available and no clear cut-off values for treatment success to optimize the treatment. Structured and quantitative follow up tools could greatly facilitate optimal care for children with ASD and provide alignment of evaluation between practices when seen by different physicians. A brief monitoring tool can only include a limited number of questions (not extensive) for follow up, and as such, the most important questions for monitoring and standardized treatment response cutoffs should be included.

### Standardizing Treatment Response Definition

**Fig. 1:**
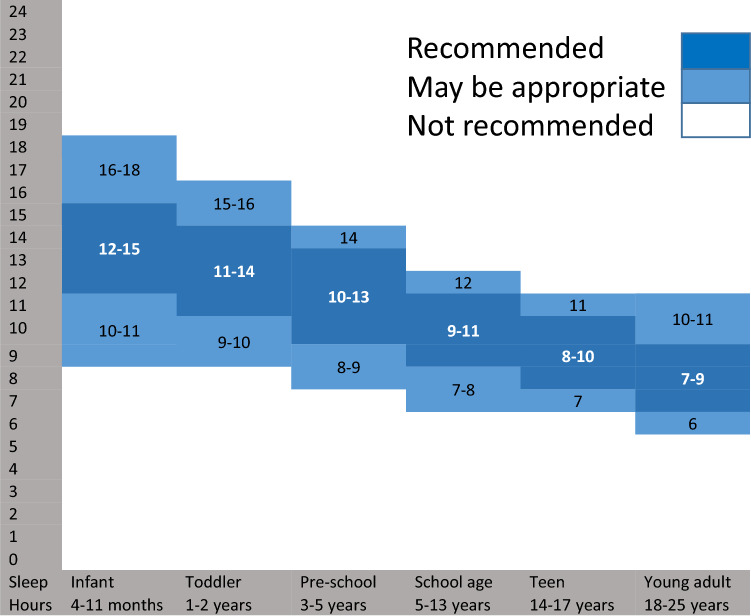
Recommended amounts of sleep for children of different ages

In line with the characteristics of insomnia in children with ASD, the goal for the purpose of treatment optimization is to achieve the following: (1) Acceptable total sleep time (TST) ranges according to the National Sleep Foundation (NSF) (Hirshkowitz et al., 2015) (Fig. 1). (2) SOL of ≤ 30 min according to DSM-5 insomnia diagnostic criteria (Cortesi et al., 2012). (3) Longest consolidated Sleep Episode (LSE, the duration of the longest uninterrupted sleep period) of > 6 h (Touchette et al., 2005; Yavuz-Kodat et al., 2020). Notably, if these cutoff values are not met, an improvement of TST and/or SOL of about 60 min in a child with ASD may indicate treatment effectiveness even if not complying fully with the recommendations (Appleton et al., 2012; Gringras et al., 2017; Sadeh, 2007). When evaluating treatment success, achieving parents’ goals (what do they want to aim at with the treatment) ought to be considered in the decision making process as well (McConachie et al., 2018). Therefore, further to quantitative (TST, SOL, LSE) measures the clinician should also evaluate improvement in the child’s behavior and parents’ satisfaction.

The structured insomnia monitoring tool proposed here by the expert panel (Table 3) was specifically designed to meet the aforementioned criteria: evaluating the child’s sleep parameters along with a personalized evaluation of behavioral features and parents’ satisfaction. It can optionally be filled by the parent and has defined cutoffs in order to enable treatment optimization by the clinician. It can also be performed at baseline, immediately after screening, in addition to the suggested screening tool, before the start of any intervention to facilitate follow up on the treatment effectiveness and measure change. It is a combined tool with a systematic assessment of important sleep parameters, along with a personalized evaluation of behavioral features based on a child’s specific clinical evaluation and parents' satisfaction to evaluate the entire scope of treatment outcomes. Not the entire behavioral checklist needs to be followed; only the specific challenging behavior/s identified by the parents and the physician at screening should be monitored during treatment to assess treatment success.

**Table 3:**
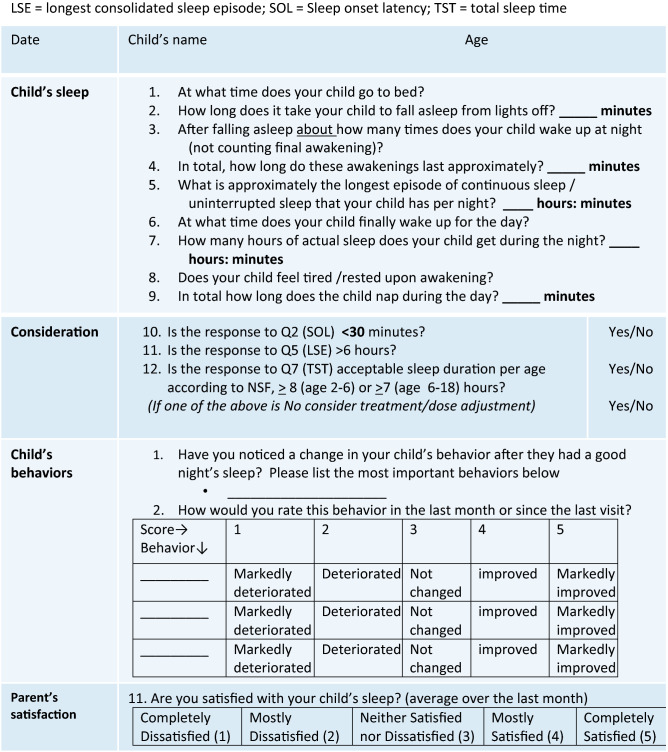
A novel structured follow up tool for insomnia-sleep

## Conclusions

The insomnia practice tools recommended herein, namely a screening tool as well as a structured monitoring tool for treatment evaluation, are short and simple tools that may help health care professionals to identify and manage insomnia in children and adolescents with ASD.

A partial limitation of the study should be noted. These tools either the screening tool for insomnia (Table 2) and the follow-up tool (Table 3) have not yet been used in a specific study and the psychometric properties have not been validated. However, concerning the screening tool for insomnia in children and adolescents, this represents the sub-scale “Disorders of Initiating and Maintaining Sleep (DIMS)” of the Sleep Disturbance Scale for Children (SDSC) that has been evaluated in different populations showing good psychometric properties either in the original scale or in the subsequent studies in children with neurodevelopmental disabilities (Bruni et al., 1996; Marriner et al., 2017). The follow-up tool should be used only as an instrument to analyze the changes after an intervention and therefore it is not necessary to evaluate the psychometric properties.

Optimal treatment should aim at: sleep onset latency of less than thirty minutes; total sleep time within the limits of the acceptable range for their age (Fig. 1); longest sleep episode of continuous sleep of more than six hours; improvement in insomnia-sensitive behaviors, and noticeable improvement in parents' satisfaction with their child’s sleep.

These tools, based on validated pediatric insomnia tools, will help identify and manage insomnia symptoms in daily practice, in order to monitor effectiveness of non-pharmacologic and pharmacologic treatments, standardize and compare outcomes across clinical settings, and substantially assist in advancing optimal care and well-being of children with ASD and their families.
